# Temporal Trends in Patient Characteristics and Clinical Outcomes of TAVR: Over a Decade of Practice

**DOI:** 10.3390/jcm13175027

**Published:** 2024-08-25

**Authors:** Nour Karra, Amir Sharon, Eias Massalha, Paul Fefer, Elad Maor, Victor Guetta, Sagit Ben-Zekry, Rafael Kuperstein, Shlomi Matetzky, Roy Beigel, Amit Segev, Israel M. Barbash

**Affiliations:** 1 Division of Cardiology, Leviev Heart and Vascular Center, Chaim Sheba Medical Center, Tel Hashomer 52621, Israel; nour.karra@sheba.health.gov.il (N.K.); amirsharon9@gmail.com (A.S.); aias.masalha@sheba.health.gov.il (E.M.); paul.fefer@sheba.health.gov.il (P.F.); elad.maor@sheba.health.gov.il (E.M.); victor.guetta@sheba.health.gov.il (V.G.); sagit.benzekry@sheba.health.gov.il (S.B.-Z.); rafael.kuperstein@sheba.health.gov.il (R.K.); shlomi.matetzky@sheba.health.gov.il (S.M.); roy.beigel@sheba.health.gov.il (R.B.); amit.segev@sheba.health.gov.il (A.S.); 2 The Faculty of Medicine, Tel Aviv University, Tel Aviv 69978, Israel

**Keywords:** TAVR, aortic stenosis, temporal trends, low risk

## Abstract

**Background/Objective:** Transcatheter aortic valve replacement (TAVR) is indicated for severe aortic stenosis patients with a prohibitive surgical risk. However, its use has been expanding in recent years to include intermediate- and low-risk patients. Thus, registry data describing changes in patient characteristics and outcomes are needed. The aim of this study was to analyse the temporal changes in patient profiles and clinical outcomes of all-comer TAVR. **Methods:** Baseline characteristics and VARC-3 outcomes of 1632 consecutive patients undergoing TAVR between 2008 and 2021 were analysed. **Results:** The annual rate of TAVR increased from 30 procedures in 2008–2009 to 398 in 2020–2021. Over the follow-up period, patient age decreased from 85 ± 4 to 80 ± 6.8 (*p* < 0.001) and the STS score decreased from 5.9% to 2.8% (*p* < 0.001). Procedural characteristics significantly changed, representing a shift into a minimally invasive approach: adoption of local anaesthesia (none to 48%, *p* < 0.001) and preference of transfemoral access (74% in 2011–2012 vs. 94.5% in 2020–2021, *p* < 0.001). The rates of almost all procedural complications decreased, including major vascular and bleeding complications, acute kidney injury (AKI) and in-hospital heart failure. There was a striking decline in rates of complete atrioventricular block (CAVB) and the need for a permanent pacemaker (PPM). PPM rates, however, remain high (17.8%). Thirty-day and one-year mortality significantly declined to 1.8% and 8.3%, respectively. Multivariable analysis shows that AKI, bleeding and stroke are strong predictors of one-year mortality (*p* < 0.001). **Conclusions:** The TAVR procedure has changed dramatically during the last 14 years in terms of patient characteristics, procedural aspects and device maturity. These shifts have led to improved procedural safety, contributing to improved short- and long-term patient outcomes.

## 1. Introduction

Since its introduction in 2007, transcatheter aortic valve replacement (TAVR) has maintained its status as an effective and safe treatment for severe aortic stenosis (AS) in patients with a high-risk surgical profile [1,2,3,4]. In recent years, evidence has accumulated to support the use of TAVR in intermediate-risk patients with severe AS, and it was proven to be at least non-inferior to surgical aortic valve replacement (SAVR) [5,6]. Notably, the PARTNER 3 and Evolut Low Risk trials have both demonstrated TAVR to be non-inferior to SAVR also in low-risk patients at 2-year follow-up [7,8,9]. Thus, the percutaneous technology has gained popularity in the treatment of patients across the entire clinical and surgical risk spectrum. 

With the constantly expanding indications of TAVR, it is no longer considered a salvage procedure but is rather becoming the standard of care for severe AS. These shifting trends are attributable to several factors: The technological advances in equipment and the development of a large range of bioprosthetic valves have made TAVR both easier and safer. Interventional cardiologists have gained experience in technique implementation and established confidence in the selection of appropriate patients. 

While RCTs no doubt provide the highest level of evidence used for practice guidelines, analysis of all-comer registry data is of paramount importance in elucidating the factors affecting clinical outcomes in daily life settings. In this regard, considering the evolving paradigms and trends in the application of TAVR, as well as the constant technological advances in the field, there is a growing need to specifically assess temporal changes in practice and patient outcomes. However, only few reports from national TAVR registries have been published so far [10,11,12,13,14,15,16]. Even fewer studies have been reported from Israel [17,18,19]. Thus, the aim of the present study was to assess all-comer TAVR trends in patient and procedural characteristics, as well as outcomes over a period span of 14 years.

## 2. Methods

This retrospective analysis included all-comer consecutive patients with severe aortic stenosis undergoing TAVR at Sheba Medical Center between the years 2008 and 2021. Data were collected prospectively and analysed retrospectively. 

Inclusion criteria consisted of all patients with severe symptomatic AS diagnosed clinically and confirmed by Doppler echocardiography who were referred for TAVR due to increased surgical risk as assessed by the institutional Heart Team. Patients underwent a complete Doppler echocardiography and computed tomographic angiography (CTA) prior to TAVR. Valve implantation was performed using either a self-expandable valve (Evolute Pro (Medtronic, Minneapolis, MN, USA), ACURATE neo (Boston Scientific, Marlborough, MA, USA), CoreValve (Medtronic, Minneapolis, MN, USA), Portico (Abbott, Lake County, IL, USA), Navitor (Abbott, Lake County, IL, USA)) or a balloon-expandable valve (Sapien-3, Sapien XT, Edwards Lifesciences, Irvine, CA, USA), according to operator discretion. 

Baseline data regarding past medical history were recorded by a blinded investigator into a computerised database. History of ischaemic heart disease, hypertension, diabetes mellitus and stroke as well as other comorbidities were extracted from patients’ electronic medical records. TAVR device success and in-hospital postprocedural complications were recorded according to the Valve Academic Research Consortium-3 (VARC-3) [20]. No patients were excluded from the analysis. Mortality rates were ascertained with the Israeli Ministry of Internal Affairs Population Registry. 

This study complied with the standards of the Helsinki Declaration and was approved by the Sheba institutional ethical committee.

Patients were divided according to procedural year: 2008–2009, 2010–2011, 2012–2013, 2014–2015, 2016–2017, 2018–2019 and 2020–2021. Data are presented according to this categorisation. The temporal changes in patients’ baseline characteristics and TAVR outcomes were analysed. 

Statistical analysis was carried out using IBM SPSS Statistics version 25 (Chicago, IL, USA) and R Project for Statistical Computing version 4.4.0. Continuous variables are presented as mean ± SD and were compared using the 1-way ANOVA test. Categorical variables are presented as numbers and percentages within years and were compared using the χ^2^ test. Kaplan–Meier was used for survival analysis, and Cox regression was used for multivariable analysis. The Log rank and the HR are given. The effects of the baseline STS score and important complications on mortality were evaluated with multivariable Cox proportional hazards models (specifically we adjusted mortality for STS, stage 2–3 AKI, major bleeding and stroke). The proportional hazards assumption was tested using the Cox proportional hazards model in R Software version 4.4.0, performing the Schoenfeld residuals test for each variable and for the overall model. A *p* value below 0.05 was considered significant. 

## 3. Results 

### 3.1. Patient Characteristics

From 2008 to 2021, a total of 1632 consecutive patients underwent TAVR and were prospectively included in this study. The annual rate of TAVR has increased constantly, from 30 procedures in 2008–2009 to 398 procedures in 2020–2021 (*p* < 0.05). The mean age of patients enrolled was 81.5 (±7.57) years, and 797 (48.9%) were males.

Throughout the study period, the percentage of women and men undergoing TAVR was relatively constant (*p* = 0.119). Table 1 presents patients’ baseline characteristics over time. Patients’ average age has gradually decreased, from 85 ± 4 at 2008–2009 to 80 ± 6.8 in 2020–2021 (*p* < 0.001).

While the burden of some comorbidities such as diabetes and dialysis-dependent CKD (chronic kidney disease) remained relatively constant throughout the years, the burden of other comorbidities such as hypertension and COPD has decreased (*p* < 0.001 for trend). It is also notable that the burden of CAD and PVD has decreased (*p* < 0.001 and *p* < 0.01 for trend, respectively), while prevalence of atrial fibrillation remained unchanged (*p* = 0.325, Table 1). Consistent with the decreasing age and burden of comorbidities, the STS (Society of Thoracic Surgeons) score decreased from 5.9% (±4.4%) to 2.8% (±3.16%) (*p* < 0.001) and the EuroScore from 4.5% (±3.1%) to 2.7% (±2.43%) (*p* < 0.05), where the most significant decrease occurred in the last four years of the follow-up period (2018–2021) (Table 1). Additionally, over the years, the baseline peak and mean pressure gradients on the treated aortic valve have also significantly decreased from 87/55 mmHg on average in 2008–2009 to 67/42 mmHg in 2020–2021 (*p* < 0.005 for trend). This is also mirrored by the increase in the average aortic valve area (AVA) from 0.63 ± 0.16 cm^2^ in 2008–2009 to 0.73 ± 0.2 cm^2^ in 2020–2021 (*p* < 0.01 for trend). 

### 3.2. Procedural Characteristics

While up to 46% of patients underwent TAVR using general anaesthesia during the earlier years of this study (2008–2011), its use has dropped significantly to only 2.5% since 2018. Parallel to this change, almost 50% of patients have undergone the procedure without any anaesthesia or sedation since 2018. Conscious sedation was still in practice in 2021, though its use has also significantly declined when compared to the earlier years, and the vast majority of procedures are now performed with local anaesthesia only (Table 2). 

Transfemoral vascular access was the dominant access site throughout the entire study period, accounting for 96% of procedures. The use of transapical access decreased from 19% to 0.4%. Beginning in 2018, transcaval access emerged as the principal alternative for patients unsuitable for transfemoral access, accounting for 2–5% of procedures (Table 2). The experience with direct aortic approach is scarce. 

As to the valve types used, in the earlier years and until 2011, CoreValve was the predominant device. From 2011 onwards, SAPIEN gained popularity, first with the XT model and from 2014 with the SAPIEN 3. In total, the large majority of valves used were CoreValve/Evolute, followed by SAPIEN. ACURATE Neo valve and Portico/Navitor valves account for only a minority of procedures, emerging in recent years (Table 2). 

Utilisation of balloon pre-dilatation became less frequent in recent years and was performed in around 60% of patients in the last quartile versus 85–90% of patients in the first quartile. 

Though valve-in-valve (VIV) procedures became more common, reaching about 6% of procedures in the last quartile, this did not reach statistical significance.

### 3.3. Outcomes 

Procedural technical success according to the VARC-3 definition [20] was achieved in more than 95% of patients, regardless of TAVR year (Table 3). Minimal and mild paravalvular leak (PVL) rates declined consistently, while the “no leak” rates increased. A need for a second valve was recorded for 2.5–6%; although there was a declining trend for this outcome, no statistically significant change was noted. The need for a second valve was mirrored by the rates of valve mal-positioning and valve migration/embolisation, which consistently and significantly decreased throughout the years (Table 3). Indeed, when reviewing the raw data, the need for a second valve coincided in most cases with valve mal-positioning and/or migration. Only rare cases of a second valve implantation (two) were due to annular rupture or moderate–severe PVL.

### 3.4. Procedure Complications

Trends in procedural complications are detailed in Table 3. Vascular and non-vascular access-related complications consistently and significantly went down from 2008 to 2021 (from 14.3% to 2.8%, *p* value < 0.001). This was true for both minor and major complications. Similarly, an evident decrease in major and life-threating bleeding events (type 2–4) was recorded. AKI rates also significantly declined (stage 1 AKI declined from 24% to 7.6%, *p* < 0.001) (Table 3). 

When cardiovascular-related complications were reviewed, in-hospital heart failure was a major sequela in the early years of TAVR, but its rate declined by more than half in 10 years. Cardiac tamponade, a potentially fatal complication, has become a rare event, occurring in 1.5% of cases in 2020–2021. Even though the rate of stroke did not significantly change over the years, it is still documented in a small minority of patients (1.8–4.3%, *p* value = 0.314). 

**Figure 1:**
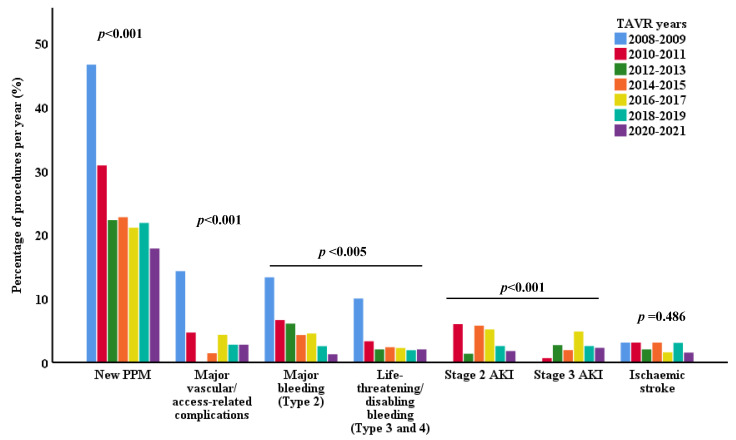
TAVR complication rates per year. Results are expressed as frequencies (N) and percentages (%) of yearly procedures. The χ^2^ test was used to compare percentages of categorical variables. *p* < 0.05 was considered significant.

As to conduction-related complications, there was a striking decline in the rates of complete atrioventricular block (CAVB) and subsequent need for a permanent pacemaker (PPM); however, PPM implantation (PPMI) rates in recent years still remain high at 17.8% (Figure 1). The rates of first-degree and second-degree AVB slightly went up, and left bundle branch block (LBBB) rates remained rather unchanged (Table 3).

### 3.5. Mortality

**Figure 2:**
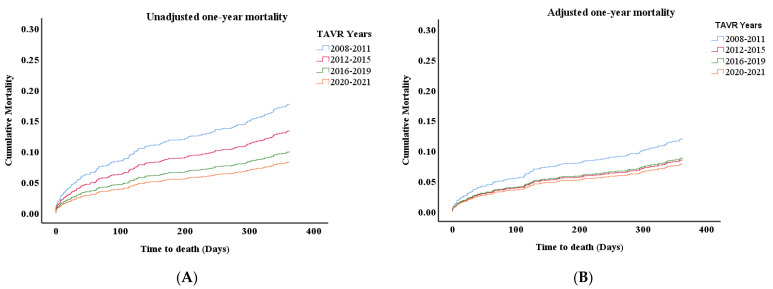
(**A**) Unadjusted cumulative probability of death over a one-year follow-up (*p* < 0.05) (**B**) Cumulative probability of death over one year adjusted for STS score, stage 2–3 AKI, major bleeding and stroke.

Thirty-day mortality significantly decreased from 5–10% in 2008–2013 to ~2% in the recent years of follow-up (Table 3, Supplementary data Figure S1 and Table S1). One-year mortality was also lower in the last years of the study period, though this difference was less pronounced. This trend of reduced mortality was preserved throughout two years of follow-up (Table 3). There was a statistically significant difference in the HR for one-year mortality with regards to TAVR years (‘’unadjusted’’ mortality), and the HR decreased as the years advanced (Figure 2A, *p* < 0.05, Table 4A). However, when adjusting cumulative mortality for the STS score and for major complications (AKI, bleeding, stroke (Figure 2B, Table 4B)), differences in mortality between years become less evident. AKI, major bleeding and stroke are important contributors which significantly increase the HR for death (Table 4B). 

## 4. Discussion

The present study presents contemporary analysis of patients’ characteristics and outcomes in all-comer TAVR procedures performed between the years 2008 and 2021. The main study findings demonstrate a decrease in patients’ age and in comorbidities, represented by a significantly lower surgical risk score during the study period. Additionally, with the advancing years, better TAVR outcomes are achieved with less valve mal-positioning and embolisation and with lower rates of almost all categories of complications. Short- and long-term mortality significantly decreased.

The data suggest TAVR patients in recent years are generally younger than in the earlier period. A similar trend was previously reported by the STS-ACC TVT registry in the US, where patients’ median age decreased from 84 to 80 in 2019 [10], and by Frydman et al. in a single centre in Israel [19]. This is in contrast to results reported by European registries [11,12,14]. However, the mean age is still averaged at 80, compatible with most recent clinical practice guidelines [4]. In line with other studies, a prominent decrease in patients’ STS score and EuroScore was recorded [10,11,12,14,15,17,18,19]. Interestingly, from 2018 onwards, the average STS score is even lower than 4%. This risk score reduction is also reflected by a decrease in some significant comorbidities, primarily hypertension, COPD, PVD and CAD. This trend, also reported by other studies [10,11,14,15] could be attributed to several factors: accumulating data from prospective randomised studies comparing TAVR with surgical approach and the established experience and success of TAVR in high-risk patients have resulted in growing confidence of interventional cardiologists in the procedure. Thus, cardiologists could be challenging the notion that TAVR should be reserved for older or high-risk patients by exhibiting the relative ease and non-inferiority of TAVR when compared to SAVR in lower-risk patients. Indeed, pivot trials such as the Evolut Low Risk study and the PARTNER-2/3 trials have provided supporting evidence for this approach [5,6,7,8]. Whether these studies actually serve as an incentive to the changing trends or rather reflect the general desire of patients and treating physicians to extend TAVR applications is debatable. 

An important trend shown in the present analysis is that although aortic valve (AV) gradients measured in TTE are still defined as severe AS, the average peak and pressure gradients are in fact lower in recent years than gradients documented in earlier years. Consistent with lower AV gradients, aortic valve area (AVA) increased from 0.63 to 0.73 cm^2^. This observation was also noted in the FRANCE TAVI study [14]. A potential explanation for such an observation may be the increased awareness and closer surveillance of AS patients, leading to an earlier intervention. Notably, a similar shift in AVA was recorded between the early high-risk RCTs (AVA = 0.6 ± 0.2 cm^2^, 0.7 ± 0.2cm^2^) [1,2,3] and the low-risk RCTs that followed in 2019 (0.8 ± 0.2 cm^2^) [7,8].

Significant changes in procedural characteristics were noted, mostly representing the shift into a minimally invasive approach, with declining rates of anaesthesia to local anaesthesia, utilisation of a transfemoral approach and less use of balloon pre-dilatation. This observation is not unique to our cohort and has also been reported by the FRANCE TAVI investigators in the STS-ACC TVT registry [10,14] and by previous works from Israel [17,19]. This is largely driven by the downsizing of device sheaths as well as by the gained technical expertise of cardiologists. For the same reasons, transfemoral access is by far the preferred access, also adopted by most physicians [10,14,15]. Balloon pre-dilatation gradually but significantly decreased, also consistent with other registries [15,19]. All these changes reflect a larger approach of simplifying TAVR as much as possible, converting it into a straightforward and safer procedure. 

In the present study, rates of almost all procedural complications decreased over the years, including vascular and bleeding complications. Similar outcomes were reported by other studies from Israel [17,19]. These changes are most likely attributed to the smaller sheaths used but also to the learning curve of the operators. A notable decrease in acute kidney injury (AKI) rates was noted, similar to results from the FRANCE TAVI, STS-ACC TVT and Swiss registries [10,14,15]. This could be related to patients’ higher baseline glomerular filtration rate (GFR) in later years (*p* < 0.001, Table 1) but could also be attributed to other procedural factors such as a shorter fluoroscopy time and lower rates of hypoperfusion injury (for example, resulting from serious bleeding). 

Pacemaker rates after TAVR decreased during the study period; however, even in recent years, the rates are high at nearly 18%. A similar incidence of 17.4% new PPMI at 1-month follow-up was found in the Evolut Low Risk trial [8]. The rate of new-onset LBBB was 20% in the most recent trials [7], and a similar incidence was observed in our data (25%, Table 3). There are conflicting reports regarding overall percentages and temporal changes in PPMI rates; while some studies are in line with our findings [11,16,17,19], others have shown stable or even increasing PPM implantation rates [12,14,15]. It is difficult to track the specific reasons responsible for the disparities between registries. However, it has been well documented that the risk for PPMI is affected by valve type and platform, implantation depth and diameter, and patients’ EuroScore risk and pre-existing conduction abnormalities [21,22]. While several technical modifications were introduced along the years with regard to valve design and implantation technique, pacemaker rates are still unacceptably high, especially when sliding into the younger patient population. Moreover, even though our results show a decrease in PPMI rates, we cannot assume this trend will be maintained over the upcoming years. There is a need to identify, risk stratify and improve the procedure in order to further lower pacemaker rates.

Though a trend towards more VIV procedures was recorded, with rates of 6–6.8% in recent years, it did not reach statistical significance, probably due to the small sample size. Similar VIV rates were reported in the STS-ACC TVT registry [10]. This early experience is expected to increase dramatically within the next decade, as there is marked increase in the utilisation of bioprostheses as compared to mechanical valves during surgery [23,24,25]. Furthermore, the growing use of TAVR as a mainstream procedure in younger patients is bound to result in more redo valve-in-valve procedures. 

Thirty-day and one-year mortality significantly declined over the follow-up period, a finding consistent with previous reports [10,11,12,14,15,16,19]. Rates of mortality at thirty days and one year recorded for 2018–2019 procedures were 2.2% and 9%, respectively, and were comparable to rates reported in Germany, France, Switzerland and Denmark [11,12,15,16]. It is no surprise that younger and less morbid TAVR patients die less. However, the significantly reduced mortality cannot be explained only by lower age and STS score; the TAVR procedure has dramatically evolved over the years, becoming simpler and safer, thus resulting in a much lower rate of complications. Indeed, when one-year mortality is adjusted for both STS score and post-procedural in-hospital complications, the differences in mortality curves become less significant. Given the findings of the multivariable analysis, it is reasonable to suggest that the decrease in mortality during the study period is not related merely to the period in time of the procedure but mostly to the increased safety of the procedure; i.e., the decrease in major complications (such as AKI) directly contributed to the improved patient outcomes.

Finally, while most of our experience and recommendations regarding TAVR rely on European and US-based RCTs, there are only scarce studies evaluating TAVR trends and practices in Middle Eastern populations and specifically in Israel; Landes and colleagues have reported an observational study from three high-volume Israeli centres, reviewing TAVR temporal trends up till 2014 [17]. Frydman et al. have reported a single-centre observational study in Israel [19]. These two works exhibited similar trends to those shown in our report, both in terms of patient characteristics and outcomes, albeit at different magnitudes. In the absence of large-scale RCTs in the Middle East, our work offers unique insights into the Middle Eastern population and thus valuable all-comer registry-based data showing similar outcomes in this population compared to major European and US randomised trials, as detailed earlier.

### Study Limitations

This is an observational study with all the downsides and limitations of a retrospective analysis, including the inability to prove causality between parameters and outcomes. At best, associations can be examined. Furthermore, it is a single-centre study. Thus, all parameters are site-reported, with no external validation. However, the reported cohort included all patients referred for TAVR without any exclusion, thus representing all-comer data. Clinically important trends could be underpowered by the small sample size, particularly where data are lacking. 

Having said that, data were collected prospectively, and data completeness in this report is rather satisfactory. Of note, many reported outcomes here are comparable to European registry results, as detailed earlier. Despite their limitations and the inability to generalise results from specific populations, registries of all-comer patients enable us to capture real-life trends as they occur.

## 5. Conclusions

The TAVR procedure has changed dramatically during the last 14 years in terms of patient characteristics, procedural aspects and device maturity. These shifts have led to improved procedural safety, contributing to improved short- and long-term patient outcomes. Additional efforts to improve this procedure may further expand its availability to broader patient populations.

## Figures and Tables

**Table 1:** Patients’ baseline characteristics by year.

Years	2008–2009	2010–2011	2012–2013	2014–2015	2016–2017	2018–2019	2020–2021
Number of patients	30	151	148	211	327	366	396
Age (years)	85.1 ± 4	83.4 ± 6.9	82.3 ± 7.9	82.2 ± 7.4	82.6 ± 7.4	80.3 ± 8.5	80.1 ± 6.8
Male, N (%)	10 (33%)	71 (47%)	68 (45.9%)	110 (52%)	148 (45%)	176 (48%)	214 (54%)
STS score (%)	5.9 ± 4.4	5.7 ± 3.37	5.9 ± 4.5	4.4 ± 2.4	4.2 ± 2.85	3.1 ± 2.16	2.8 ± 3.16
EuroScore II (%)	4.5 ± 3.1	6.2 ± 6.63	5.5 ± 6.2	5.4 ± 6.8	4.1 ± 4.47	4.6 ± 22.54	2.7 ± 2.43
LVEF (%)	59.6 + 7.8	53 ± 13.4	54.6 ± 13.2	53.9 ± 12.7	55.9 ± 11.8	55 ± 12.1	54.8 ± 12
AV peak PG (mmHg) *	87.3 ± 22.7	75.3 ± 25.4	70.8 ± 23.3	66.7 ± 23.3	66.9 ± 23.2	70.9 ± 48.3	67.5 ± 21
AV mean PG (mmHg) *	55.4 ± 15.1	46.1 ± 15.6	45 ± 18.2	42.9 ± 19.4	42 ± 15.1	42.6 ± 15	42.3 ± 13.9
AVA (cm^2^) *	0.63 ± 0.16	0.69 ± 0.18	0.71 ± 0.21	0.74 ± 0.23	0.71 ± 0.18	0.74 ± 0.19	0.73 ± 0.2
BMI	27.2 ± 4.1	28.2 ± 12.7	27.2 ± 4.4	28 ± 5.4	27.9 ± 5.3	28.3 ± 5.3	28.8 ± 12
Hypertension, N (%)	26 (86.7%)	128 (84.8%)	132 (89.2%)	165 (78.6%)	254 (78.4%)	246 (77%)	283 (71.6%)
GFR (ml/min)	50 ± 15.9	50.4 ± 17.1	49.7 ± 19.5	58 ± 21.1	64 ± 23.5	66.5 ± 25.4	62.7 ± 24.3
Dialysis, N (%)	0	2 (1.3%)	3 (2.1%)	5 (2.4%)	7 (2.2%)	4 (1.3%)	13 (3.3%)
Diabetes, N (%)	12 (40%)	52 (34.7%)	56 (38%)	94 (44.8%)	118 (36.6%)	121 (38.3%)	160 (40.5%)
COPD, N (%)	2 (6.7%)	25 (16.8%)	27 (18.4%)	33 (15.7%)	19 (5.9%)	19 (6%)	23 (5.9%)
PVD, N (%)	3 (10%)	24 (16.2%)	21 (14.4%)	26 (12.3%)	49 (15.2%)	24 (9.4%)	27 (6.9%)
CAD, N (%)	15 (50%)	91 (61%)	69 (47%)	103(48.8%)	193 (59.8%)	151 (47.8%)	173 (44.5%)
Previous MI, N (%) *	6 (20%)	42 (28.6%)	45 (31.3%)	40 (19%)	98 (31%)	26 (15.4%)	36 (20.9%)
Previous CABG, N (%) *	8 (26.7%)	39 (26.2%)	28 (19.4%)	42 (20%)	78 (24.7%)	79 (45.7%)	53 (31%)
Previous stroke, N (%)	5 (16.7%)	20 (13.5%)	26 (17.7%)	33 (15.6%)	52 (16%)	26 (8.2%)	56 (14.3%)
History of AF, N (%)	8 (26.7%)	40 (26.7%)	40 (27.4%)	74 (35.2%)	94 (29%)	93 (27.8%)	101(25.5%)

Results of numerical parameters are expressed as mean ± SD. Results of categorical parameters are expressed as frequencies (N) and percentages (%). ANOVA test was used to compare numerical variables across the year groups, and the χ^2^ test was used to compare percentages of categorical variables. * denotes analysis performed for parameters with some missing data. STS—Society of Thoracic Surgeons, LVEF—left ventricular ejection fraction, AV—aortic valve, PG—pressure gradient, AVA—aortic valve area, BMI—body mass index, GFR—glomerular filtration rate, COPD—chronic obstructive pulmonary disease, PVD—peripheral vascular disease, CAD—coronary artery disease, MI—myocardial infarction, CABG—coronary artery bypass graft, AF—atrial fibrillation.

**Table 2:** Procedure characteristics by year.

Years	2008–2009	2010–2011	2012–2013	2014–2015	2016–2017	2018–2019	2020–2021	*p* Value
Number of Patients	30	151	148	211	327	366	396	
Vascular access								
Transfemoral	29 (96.7%)	112 (74.2%)	111 (75%)	167 (79%)	301 (92.3%)	351 (96.4%)	376(94.5%)	<0.001
Axillary	1 (3.3%)	8 (5.3%)	10 (6.8%)	8 (3.8%)	5 (1.5%)	1 (0.3%)	1 (0.3%)	
Transapical	0	29 (19.2%)	27 (18.2%)	36 (17%)	20 (6.1%)	5 (1.4%)	1 (0.3%)	
Direct aortic	0	2 (1.3%)	0	0	0	0	0	
Transcaval	0	0	0	0	0	7 (1.9%)	20 (5%)	
Anaesthesia method								
General sedation	6 (37.5%)	69 (46.3%)	44 (29.7%)	53 (25%)	29 (8.9%)	9 (2.5%)	10 (2.5%)	<0.001
Conscious sedation	10 (62.5%)	80 (53.7%)	104 (70.3%)	152 (72%)	290 (88.7%)	229 (62.7%)	191(48%)	
Conscious converted to general sedation	0	0	0	6 (2.8%)	7 (2.1%)	4 (1.1%)	5 (1.3%)	
No sedation (local only)	0	0	0	0	1 (0.3%)	123 (33.7%)	191 (48%)	
Balloon pre-dilatation	25 (83.3%)	141 (93.4%)	125 (85%)	83 (41.3%)	142 (43.7%)	158 (43.4%)	242 (61%)	<0.001
Balloon post-dilatation	2 (6.7%)	29 (19.6%)	26 (17.9%)	45 (22.6%)	96 (29.4%)	104 (28.5%)	82 (20.8%)	<0.005
Valve-in-valve (VIV)	0	5 (3.3%)	7 (4.7%)	9 (4.3%)	21 (6.5%)	22 (6%)	27 (6.8%)	0.442
Valve type								
CoreValve/Evolute	30 (100%)	92 (61.3%)	74 (50.3%)	96 (45.9%)	195 (59.8%)	186 (51%)	151 (41.6%)	<0.001
SAPIEN	0	58 (38.7%)	73 (49.7%)	112 (53.6%)	120 (36.8%)	128 (35%)	165(45.5%)	
ACURATE Neo	0	0	0	0	0	44 (12%)	38 (10.5%)	
Portico/Navitor	0	0	0	1 (0.5%)	11 (3.4%)	7 (1.9%)	9 (2.5%)	

Results of categorical parameters are expressed as frequencies (N) and percentages (%). The χ^2^ test was used to compare percentages.

**Table 3:** TAVR outcomes and in-hospital peri-procedural complications by year.

Years	2008–2009	2010–2011	2012–2013	2014–2015	2016–2017	2018–2019	2020–2021	*p* Value
Number of Patients	30	151	148	211	327	366	396	
TAVR device success rate % (VARC-3)	100%	98%	97.3%	96.6%	97.5%	95%	95%	0.261
Need for a second valve	1 (3.3%)	7 (4.6%)	9 (6.2%)	12 (5.9%)	8 (2.5%)	13 (3.5%)	9 (2.3%)	0.150
Final perivalvular leak (PVL) (per angiography)								
None	4 (13.3%)	44 (29.3%)	60(42.3%)	88(45.6%)	96 (29.9%)	143 (39.2%)	195(49.2%)	<0.001
Minimal	16(53.3%)	67 (44.7%)	62 (43.7%)	67 (34.7%)	109(34%)	127 (34.8%)	123 (31%)	
Mild	10 (33.3%)	38 (25.3%)	17 (12%)	33 (17%)	103 (32%)	82 (22.5%)	76 (19.2%)	
Moderate	0	1 (0.7%)	1 (0.7%)	4 (2.1%)	11 (3.4%)	13 (3.6%)	2 (0.5%)	
Severe	0	0	2 (1.4%)	1 (0.5%)	2 (0.6%)	0	0	
Vascular or access related non-vascular complication (VARC-3)								
Major	4 (14.3%)	7 (4.7%)	0	3 (1.4%)	14 (4.3%)	10 (2.8%)	11 (2.8%)	<0.001
Minor	8 (28.6%)	22 (14.7%)	37 (25.2%)	51 (24.3%)	67 (20.6%)	85 (23.5%)	69 (17.3%)	
Bleeding								
Bleeding minor (Type 1)	3 (10%)	16 (10.6%)	16 (10.8%)	29 (13.8%)	37 (12%)	54 (17%)	45 (11.4%)	<0.005
Bleeding major (Type 2)	4 (13.3%)	10 (6.6%)	9 (6%)	9 (4.3%)	14 (4.5%)	8 (2.5%)	5 (1.3%)	
Life-threatening/disabling (Type 3–4)	3 (10%)	5 (3.3%)	3 (2%)	5 (2.4%)	7 (2.3%)	6 (1.9%)	8 (2%)	
Stroke								
Ischaemic stroke/TIA	1 (3.3%)	6 (4%)	4 (2.7%)	9 (4.3%)	5 (1.6%)	13 (4%)	7 (1.8%)	0.314
Haemorrhagic stroke	0	0	0	1 (0.5%)	0	0	0	
New AVB								
First-degree AVB *	1 (10%)	0	1 (3%)	1 (1.5%)	1 (0.6%)	21 (6.7%)	46 (11.7%)	<0.001
Second-degree AVB *	0	0	0	0	3 (1.8%)	3 (1%)	8 (2%)	
CAVB *	8 (80%)	26 (57.8%)	17 (51.5%)	33 (49.3%)	50 (29.6%)	50 (15.9%)	47 (11.9%)	
New permanent PPM	14 (46.7%)	46(30.9%)	33 (22.3%)	48 (22.7%)	69 (21%)	80 (21.9%)	71 (17.8%)	<0.001
New LBBB	8 (27.6%)	34 (23%)	36 (24.3%)	54 (25.7%)	83 (25.5%)	78 (21.8%)	101(25.6%)	0.883
AKI (VARC-3)								
Stage 1	7 (24%)	28 (18.7%)	25 (16.9%)	28 (13.4%)	22 (7%)	34 (10.8%)	30 (7.6%)	<0.001
Stage 2	0	9 (6%)	2 (1.4%)	12 (5.7%)	16 (5.2%)	8 (2.5%)	7 (1.8%)	
Stage 3	0	1 (0.7%)	4 (2.7%)	4 (1.9%)	15 (4.8%)	8 (2.5%)	9 (2.3%)	
Cardiac tamponade	3 (10%)	5 (3.3%)	3 (2%)	3 (1.4%)	5 (1.5%)	4 (1.1%)	6 (1.5%)	<0.05
Conversion to open surgery	0	3 (2%)	0	2 (0.9%)	3 (0.9%)	2 (0.5%)	1 (0.3%)	0.338
Valve mal-position	1 (3.3%)	9 (6%)	6 (4.1%)	7 (3.3%)	2 (0.6%)	2 (0.5%)	4 (1%)	<0.001
Valve migration/embolisation	1 (3.3%)	5 (3.3%)	5 (3.4%)	6 (2.8%)	3 (0.9%)	1 (0.3%)	3 (0.8%)	<0.05
Peri-procedure MI (till 72 h)	0	3 (2%)	7 (4.7%)	1 (0.5%)	4 (1.2%)	1 (0.3%)	8 (2%)	<0.01
In-hospital HF	10 (34.5%)	21 (14.8%)	12 (8.2%)	32 (15.6%)	16 (5.2%)	22 (7%)	16 (4%)	<0.001
Days from TAVR till discharge	5.99 [4.9–6.2]	5.99 [4.9–9.9]	5.9 [3.9–7.9]	4.9 [3.9–7.9]	3.9 [2.9–5.9]	3.9 [2.9–5.9]	3.9 [2.9–5.9]	-
Mortality								
Thirty-day mortality	3 (10%)	7 (4.6%)	8 (5.4%)	5 (2.4%)	11 (3.4%)	8 (2.2%)	7 (1.8%)	<0.05
One-year mortality	3 (10%)	29 (19.2%)	20 (13.5%)	28 (13.3%)	36 (11%)	33 (9%)	33 (8.3%)	<0.01
Two-year mortality	6 (20%)	43 (28.5%)	29 (19.6%)	50 (23.7%)	61 (18.7%)	66 (18%)	72 (18.1%)	0.095

Results are expressed as frequencies (N) and percentages (%). The χ^2^ test was used to compare percentages of categorical variables. Days till discharge are presented as median (IQR [CI]). TIA—transient ischaemic attack, AKI—acute kidney injury, HF—heart failure, LBBB—left bundle branch block, CAVB—complete atrioventricular block, PPM—permanent pacemaker. * denotes analysis performed for parameters with some missing data.

**Table 4:** Unadjusted (A) and adjusted (B) hazard ratios (HR) of death over a follow-up period of one year. One-year mortality was adjusted for STS score, stage 2-3 AKI, major bleeding and stroke (4B).

**(A)**	**HR**	**95% CI**	***p* Value**
2008–2011	Reference	Reference	Reference
2012–2015	0.736	[0.47–1.15]	0.178
2016–2019	0.537	[0.35–0.81]	0.004
2020–2021	0.444	[0.27–0.72]	0.001
**(B)**	**HR**	**95% CI**	***p* Value**
2008–2011	Reference	Reference	Reference
2012–2015	0.68	[0.41–1.12]	0.126
2016–2019	0.68	[0.44–1.07]	0.093
2020–2021	0.61	[0.37–1.01]	0.056
STS score	1.09	[1.07–1.11]	<0.001
Stage 2–3 AKI	3.42	[2.18–5.38]	<0.001
Major bleeding	2.26	[1.41–3.63]	0.001
Stroke	3.51	[1.93–6.39]	<0.001

## Data Availability

The data presented in this study are available on request from the corresponding author.

## Supplementary Materials

The following supporting information can be downloaded at: https://www.mdpi.com/article/10.3390/jcm13175027/s1, Figure S1: Unadjusted and adjusted cumulative probability of death over a follow-up period of 30 days. Probability of death was adjusted for STS score, stage 2–3 AKI, major bleeding and stroke.; Table S1: Unadjusted (A) and adjusted (B) hazard ratios (HR) of death for TAVR years over a follow-up period of 30 days. Mortality was adjusted for STS score, stage 2-3 AKI, major bleeding and stroke.
