# Supplementary material for: Temporal Trends in Patient Characteristics and Clinical Outcomes of TAVR: Over a Decade of Practice
Source: J Clin Med. 2024 Aug 25;13(17):5027. doi: 10.3390/jcm13175027 (PMC11396256; doi:10.3390/jcm13175027)
Supplement: Supplementary file 1 [file jcm-13-05027-s001.zip › jcm-3137578-supplementary.pdf]

Supplementary data

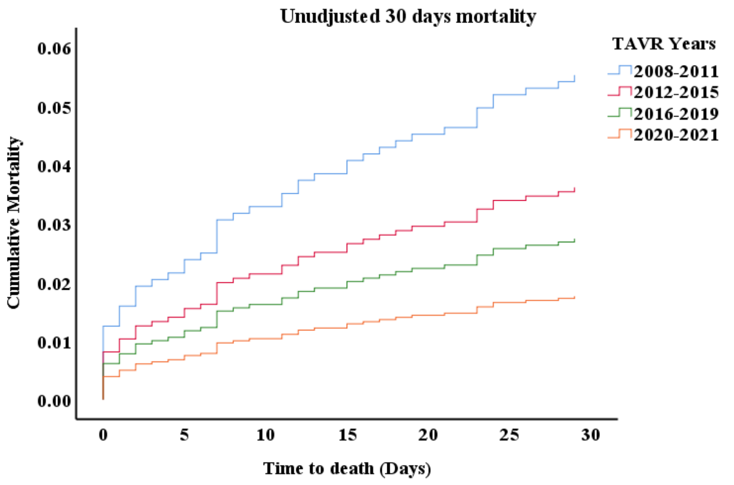

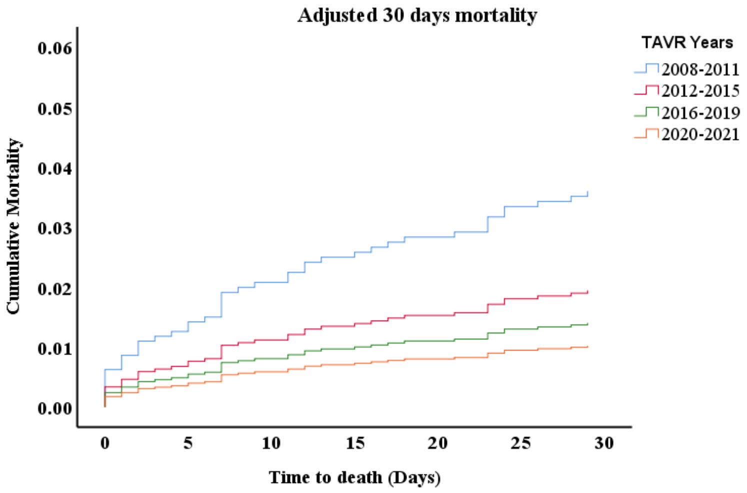

<u>Figure S1:</u> Unadjusted and adjusted cumulative probability of death over a follow-up period of 30 days. Probability of death was adjusted for STS score, stage 2–3 AKI, major bleeding and stroke.

<u>Table S1:</u> Unadjusted (A) and adjusted (B) hazard ratios (HR) of death for TAVR years over a follow-up period of 30 days. Mortality was adjusted for STS score, stage 2-3 AKI, major bleeding and stroke.

Table S1A	HR	95 % CI	*p*-value
**2008-2011**	Reference	Reference	Reference
**2012-2015**	0.647	[0.284-1.47]	.301
**2016-2019**	0.489	[0.227-1.05]	.067
**2020-2021**	0.313	[0.119-0.821]	.018

Table S1B	HR	95 % CI	*p*-value
**2008-2011**	Reference	Reference	Reference
**2012-2015**	0.536	[0.219-1.31]	0.172
**2016-2019**	0.385	[0.162-0.915]	0.031
**2020-2021**	0.280	[0.098-0.798]	0.017
**STS score**	1.1	[1.067-1.136]	0.000
**Stage 2-3 AKI**	8.29	[4.03-17]	0.000
**Major bleeding**	5.7	[2.76-11.78]	0.000
**Stroke**	5.33	[1.8-15.7]	0.002
